# Moderators of exercise effects on self-reported cognitive functioning in cancer survivors: an individual participant data meta-analysis

**DOI:** 10.1007/s11764-023-01392-3

**Published:** 2023-05-09

**Authors:** Anouk E. Hiensch, Julia Beckhaus, Lenja Witlox, Evelyn M. Monninkhof, Sanne B. Schagen, Jonna K. van Vulpen, Maike G. Sweegers, Robert U. Newton, Neil K. Aaronson, Daniel A. Galvão, Karen Steindorf, Martijn M. Stuiver, Ilse Mesters, Hans Knoop, Martine M. Goedendorp, Martin Bohus, Lene Thorsen, Karl-Heinz Schulz, Martina E. Schmidt, Cornelia M. Ulrich, Gabe S. Sonke, Wim H. van Harten, Kerri M. Winters-Stone, Miranda J. Velthuis, Dennis R. Taaffe, Willem van Mechelen, Marie José Kersten, Frans Nollet, Joachim Wiskemann, Laurien M Buffart, Anne M May

**Affiliations:** 1 Julius Center for Health Sciences and Primary Care, University Medical Center Utrecht, Utrecht University, Utrecht, The Netherlands; 2 Division of Psychosocial Research and Epidemiology, Netherlands Cancer Institute, Amsterdam, The Netherlands; 3 Department of Radiation Oncology, University Medical Center Utrecht, Utrecht University, Utrecht, The Netherlands; 4 Division of Psychosocial Research and Epidemiology & Center for Quality of Life, Netherlands Cancer Institute, Amsterdam, The Netherlands; 5 Exercise Medicine Research Institute, Edith Cowan University, Perth, WA Australia; 6 Division of Physical Activity, Prevention and Cancer, German Cancer Research Center (DKFZ) and National Center for Tumor Diseases (NCT) Heidelberg, Heidelberg, Germany; 7 Department of Epidemiology, Maastricht University, Maastricht, The Netherlands; 8 Department of Medical Psychology, Amsterdam UMC location University of Amsterdam, Amsterdam, The Netherlands; 9 Department of Health Psychology, University Medical Center Groningen, University of Groningen, Groningen, The Netherlands; 10 Department of Health Sciences, Faculty of Science, Vrije Universiteit Amsterdam, Amsterdam Public Health Research Institute, Amsterdam, The Netherlands; 11 Institute of Psychiatric and Psychosomatic Psychotherapy, Central Institute of Mental Health, Mannheim, Heidelberg University, Heidelberg, Germany; 12 Faculty of Health, University of Antwerp, Antwerp, Belgium; 13 National Advisory Unit on Late Effects after Cancer, Department of Oncology, Oslo University Hospital, Oslo, Norway; 14 Department of Clinical Service, Division of Cancer Medicine, Oslo University Hospital, Oslo, Norway; 15 Athleticum – Competence Center for Sports- and Exercise Medicine and Institute for Medical Psychology, University Medical Center Hamburg-Eppendorf, Hamburg, Germany; 16 Huntsman Cancer Institute and Department of Population Health Sciences, University of Utah, Salt Lake City, USA; 17 Netherlands Cancer Institute, Amsterdam, The Netherlands; 18 Department of Health Technology and Services Research, University of Twente, Enschede, The Netherlands; 19 Division of Oncological Sciences, Knight Cancer Institute, Oregon Health & Science University, Portland, OR USA; 20 Netherlands Comprehensive Cancer Organisation (IKNL), Utrecht, The Netherlands; 21 Department of Public and Occupational Health, Amsterdam Public Health Research Institute, Amsterdam UMC, location: Vrije Universiteit, Amsterdam, The Netherlands; 22 School of Human Movement and Nutrition Sciences, Faculty of Health and Behavioural Sciences, University of Queensland, Brisbane, Australia; 23 Division of Exercise Science and Sports Medicine (ESSM), Department of Human Biology, Faculty of Health Sciences, University of Cape Town, Cape Town, South Africa; 24 School of Public Health, Physiotherapy and Population Sciences, University College Dublin, Dublin, Ireland; 25 Department of Hematology, Amsterdam University Medical Centers, Location University of Amsterdam, Cancer Center Amsterdam, Amsterdam, The Netherlands; 26 Department of Rehabilitation Medicine, Amsterdam UMC location University of Amsterdam, Amsterdam, The Netherlands; 27 Amsterdam Movement Sciences, Rehabilitation & Development, Amsterdam, The Netherlands; 28 Working Group Exercise Oncology, Division of Medical Oncology, University Clinic Heidelberg and National Center for Tumor Diseases (NCT), Heidelberg, Germany; 29 Department of Physiology, Radboudumc, Nijmegen, The Netherlands

**Keywords:** Cancer, Cognitive functioning, Exercise, Individual participant data meta-analysis, Survivorship

## Abstract

**Purpose:**

This individual participant data meta-analysis (IPD-MA) assesses exercise effects on self-reported cognitive functioning (CF) and investigates whether effects differ by patient-, intervention-, and exercise-related characteristics.

**Methods:**

IPD from 16 exercise RCTs, including 1987 patients across multiple types of non-metastatic cancer, was pooled. A one-stage IPD-MA using linear mixed-effect models was performed to assess exercise effects on self-reported CF (*z*-score) and to identify whether the effect was moderated by sociodemographic, clinical, intervention- and exercise-related characteristics, or fatigue, depression, anxiety, and self-reported CF levels at start of the intervention (i.e., baseline). Models were adjusted for baseline CF and included a random intercept at study level to account for clustering of patients within studies. A sensitivity analysis was performed in patients who reported cognitive problems at baseline.

**Results:**

Minimal significant beneficial exercise effects on self-reported CF (β=−0.09 [−0.16; −0.02]) were observed, with slightly larger effects when the intervention was delivered post-treatment (*n*=745, β=−0.13 [−0.24; −0.02]), and no significant effect during cancer treatment (*n*=1,162, β=−0.08 [−0.18; 0.02]). Larger effects were observed in interventions of 12 weeks or shorter (β=−0.14 [−0.25; −0.04]) or 24 weeks or longer (β=−0.18 [−0.32; −0.02]), whereas no effects were observed in interventions of 12–24 weeks (β=0.01 [−0.13; 0.15]). Exercise interventions were most beneficial when provided to patients without anxiety symptoms (β=−0.10 [−0.19; −0.02]) or after completion of treatment in patients with cognitive problems (β=−0.19 [−0.31; −0.06]). No other significant moderators were identified.

**Conclusions:**

This cross-cancer IPD meta-analysis observed small beneficial exercise effects on self-reported CF when the intervention was delivered post-treatment, especially in patients who reported cognitive problems at baseline.

**Implications for Cancer Survivors:**

This study provides some evidence to support the prescription of exercise to improve cognitive functioning. Sufficiently powered trials are warranted to make more definitive recommendations and include these in the exercise guidelines for cancer survivors.

**Supplementary Information:**

The online version contains supplementary material available at 10.1007/s11764-023-01392-3.

## Introduction

After a cancer diagnosis, many patients experience short- and long-term side effects of cancer and its treatment, including fatigue, reduced physical fitness, anxiety and depression, and cognitive problems [1–4]. The prevalence of long-term (i.e., >10 years) cognitive problems among cancer survivors ranges from 15 to 70%, including problems with learning, memory, attention, and concentration [5–8]. Although cognitive problems assessed objectively or subjectively appear to be mild to moderate, such problems can have a significant impact on health-related quality of life (HRQoL), the ability to return to work and daily life activities [5, 9–12].

There is ample evidence that physical exercise during and after cancer treatment has beneficial effects on HRQoL and other relevant patient-reported outcomes such as fatigue and physical functioning [13, 14]. Results from preclinical studies suggest that exercise might be an effective strategy to reduce cancer-related cognitive problems by targeting the biological mechanisms affected by cancer treatment [15]. In particular, hippocampal neurogenesis, which is important for learning and memory functioning, seems to be improved after exercise [15, 16]. There is also strong evidence of a positive effect of exercise on cognitive problems in healthy older adults and patients with mild cognitive impairment [17, 18]. However, to date, evidence of a positive effect of exercise on cognitive functioning in cancer survivors from randomized controlled trials (RCTs) is limited and inconclusive [19].

In a recent systematic review, Campbell and colleagues reported a beneficial effect of exercise on self-reported cognitive problems in patients with different types of cancer [20]. However, only three (10%) of twenty-nine exercise intervention studies included self-reported cognitive functioning as a primary outcome. Since the other studies included self-reported cognitive functioning as a secondary outcome, they were underpowered to detect meaningful intervention effects and did not use comprehensive questionnaires to assess self-reported cognitive functioning. To date, one sufficiently powered study assessed the effects of a 6-month supervised exercise intervention on cognitive functioning in chemotherapy-exposed breast cancer patients with cognitive problems 2–4 years after completion of their cancer treatment [21]. Although no significant effects were found for objectively assessed cognitive functioning, significant positive effects were found on self-reported cognitive functioning [22].

To effectively target exercise interventions, it is important to identify which patients benefit most from specific exercise programs (i.e., identify moderators of exercise intervention effects). Two studies in breast cancer survivors showed that patients who were ≤2 years post-surgery or on endocrine therapy had a greater improvement in cognitive functioning compared to patients who were >2 years post-surgery or not on endocrine therapy, respectively [23, 24]. However, these studies were not sufficiently powered to assess moderators of intervention effects. Also, meta-analyses based on aggregated data, while useful for increasing statistical power, are prone to ecological bias when used to identify moderators, since they do not take participant-level characteristics into account [25, 26]. Therefore, a meta-analysis using individual participant data (IPD) is the preferred method for investigating potential moderators of intervention effects. By securing raw data per participant, this type of meta-analyses provides more statistical power to be able to disentangle study- and participant-level sources of heterogeneity in intervention effects [25]. Here, we report the results of an IPD meta-analysis whose aims were to assess the effect of exercise interventions on self-reported cognitive functioning and to investigate moderators of the exercise effect in patients with non-metastatic cancer.

## Methods

We used data collected in the Predicting Optimal cAncer RehabIlitation and Supportive care (POLARIS) study, an international infrastructure and shared database of RCTs investigating the effect of exercise and psychosocial interventions in patients with cancer on a range of outcomes (registered in PROSPERO, CRD42013003805). Details of the POLARIS study design, including all procedures and methods of study identification and selection, have been published previously [27]. The meta-analysis was conducted in accordance with the PRISMA guidelines [28]. All individual studies were performed in line with the principles of the Declaration of Helsinki and received approval from their local ethics committees. Informed consent was obtained from all individual participants included in the individual studies. For the current IPD meta-analysis, we included all exercise RCTs in the POLARIS database that assessed self-reported cognitive functioning (16 RCTs, *n*=1987 participants). We excluded patients with metastatic disease due to the small sample size (*n*=61).

### Quality assessment

Two authors (MGS and LB) independently assessed the methodological quality of each included RCT using the “risk of bias” tool of the Cochrane Collaboration. The following aspects were graded as high, low, or unclear quality: random sequence generation, allocation concealment, incomplete outcome, incomplete reporting, adherence, and contamination. A full quality assessment of the included studies can be found in our previous publication [14].

### Outcomes

The main outcome, self-reported cognitive functioning, was assessed using the corresponding subscale of the EORTC QLQ-C30 [29] (7 studies [30–36]), the mental fatigue subscale of the Multidimensional Fatigue Inventory (MFI) [37] (6 studies [38–44]), the concentration problems dimension of the Checklist Individual Strength (CIS) [45] (1 study [46]), or the cognitive fatigue dimension of the Fatigue Assessment Questionnaire (FAQ) [47] (2 studies [48, 49]) (Table 1). If a study used multiple questionnaires, we used data from the (1) MFI, (2) CIS, (3) FAQ, or (4) EORTC QLQ-C30. This hierarchical order is based on the number of items and multidimensionality of the questionnaire.

**Table 1:** Overview of the questionnaires used to assess self-reported cognitive functioning

Position	Questionnaire	Questions/statements
1	MFI (*5-point Likert scale*)	- When I am doing something, I can keep my thoughts on it. - I can concentrate well. - It takes a lot of effort to concentrate on things. - My thoughts easily wander.
2	CIS (*7-point Likert scale*)	- Thinking requires effort. - When I am doing something, I can keep my thoughts on it. - I find it easy to concentrate. - It takes a lot of effort to concentrate on things. - My thoughts easily wander.
3	FAQ (*4-point Likert scale*)	- Have you had trouble concentrating? - Did you feel more forgetful than usual? - Was it difficult for you to stay alert, for example when listening or reading?
4	EORTC QLQ-C30 (*4-point Likert scale*)	- Have you had difficulty in concentrating on things, like reading a newspaper or watching television? - Have you had difficulty remembering things?

*MFI* Multidimensional Fatigue Inventory, *FAQ* Fatigue Assessment Questionnaire, *EORTC QLQ-C30* European Organization for Research and Treatment of Cancer Quality of Life Questionnaire–Core 30 (cognitive functioning subscale), *CIS* checklist individual strength

### Potential moderators

The following characteristics were tested as potential moderators of exercise effects on self-reported cognitive functioning: (1) sociodemographic characteristics—age, gender, and education level (low/middle vs. high); (2) clinical characteristics—cancer type (breast, male genitourinary, hematological, gastrointestinal, gynecological vs. other) and treatment type (ever receipt of surgery, chemotherapy, radiotherapy, and/or hormonal therapy yes/no); and (3) intervention characteristics—delivery mode (supervised vs. unsupervised). We stratified a priori for intervention timing (during vs. post-cancer treatment), since in general the aim of exercise programs during cancer treatment is to prevent a decline in cognitive functioning, whereas the aim of exercise post-cancer treatment is often to improve cognitive functioning. Although preliminary studies show that aerobic training might be more effective in improving cognitive functioning [13], we were not able to examine exercise modality and intensity as potential moderators due to too little variation across studies (i.e., almost all supervised interventions included resistance training (*n*=883 (resistance training) vs. *n*=29 (no resistance training))). In addition, we assessed moderator effects of continuous baseline levels of self-reported cognitive functioning, fatigue, depression, and anxiety. See Appendix I for the questionnaires used to measure these outcomes/moderators.

### Statistical analysis

Descriptive statistics were used to summarize patient characteristics and baseline levels of potential moderators. To allow pooling of the different cognitive functioning, fatigue, and depression/anxiety questionnaires, we calculated *Z*-scores by subtracting the mean score from the individual score at baseline per questionnaire and dividing the result by the mean standard deviation at baseline per questionnaire.

We used a one-stage approach to examine effects of exercise on cognitive functioning by analyzing IPD from all trials simultaneously, while accounting for clustering of participants within studies and heterogeneity across studies by including a random intercept on study level. The models were adjusted for the baseline value of cognitive functioning. All analyses were conducted according to the intention-to-treat principle. Between-group differences in *z*-scores were reported (with corresponding 95% CI), which correspond to a Cohen’s *d* effect size (<0.2 minimal effect; 0.2–0.5 small effect; 0.5–0.8 medium effect; ≥0.8 large effect) [50].

To examine whether the effects of exercise on cognitive functioning were moderated, we extended the aforementioned model to include interaction terms of the group allocation with potential moderators. The individual values of potential moderators (i.e., sociodemographic and clinical moderators) were centered around their mean study value to avoid ecological bias for patient-level interactions. We did not center values of potential intervention- and exercise-related moderators, since these do not vary within studies. The independent variables in the models were random intercept, group allocation (exercise intervention or control group), baseline value of cognitive functioning, potential moderator, and an interaction term (potential moderator x group allocation). Potential moderators were examined one-by-one in separate models. If the likelihood ratio test indicated that the interaction term improved the model fit (*p*<0.10), we considered the characteristic to be a moderator. This *p-*value was chosen because of the hypothesis generating nature of our study. When a characteristic appeared to be a moderator, stratified analyses were performed.

If a RCT consisted of three study arms with different intervention characteristics, interaction testing for intervention-related characteristics was not possible. Therefore, this potential moderator was evaluated by using dummy variables for the intervention-related characteristic (i.e., delivery mode: supervised vs. unsupervised exercise). For all post-treatment exercise intervention studies, a sensitivity analysis was performed in patients who reported at least some cognitive problems, i.e., excluding participants with the best possible score for cognitive functioning at baseline, since we hypothesize that these patients would be most in need of an intervention for cognitive functioning. Statistical analyses were performed using IBM SPSS (Version 26.0.0.1) and R (4.0.3).

## Results

In the POLARIS database, 16 studies assessed self-reported cognitive functioning, including 1987 patients with cancer. In total, 1115 patients were randomized to an exercise intervention and 872 patients to a control group. Baseline sociodemographic and clinical characteristics are presented in Table 2. Patients were, on average, 55.4 (±12.6 (SD)) years of age and the majority (64.1%) of participants were female. The most common cancer type was breast cancer (53.4%). Baseline characteristics were balanced between the exercise intervention and control group.

**Table 2:** Patient characteristics at baseline stratified by exercise intervention and control group

	Exercise (*n*=1115)	Control (*n***=**872)
*Sociodemographic characteristics*		
Age, mean (SD) years	54.7 (12.8)	55.3 (12.2)
Male, *n* (%)	402 (36.1)	311 (35.7)
Married/living with partner, *n* (%)		
Yes	828 (74.3)	604 (69.3)
No	190 (17.0)	162 (18.6)
Unknown	97 (8.7)	106 (12.2)
Education level, *n* (%)		
Low/middle	597 (53.5)	482 (55.3)
High	414 (37.1)	282 (32.3)
Unknown	104 (9.3)	108 (12.4)
*Clinical characteristics*		
BMI, mean (SD) kg/m^2^	26.6 (4.5)	27.0 (4.7)
Cancer type, *n* (%)		
Breast	598 (53.6)	464 (53.2)
Male genitourinary	267 (23.9)	197 (22.6)
Hematological	137 (12.3)	128 (14.7)
Gastrointestinal	74 (6.6)	49 (5.6)
Gynecological	28 (2.5)	25 (2.9)
Other	11 (1.0)	9 (1.0)
Surgery, *n* (%)		
Yes	797 (71.5)	607 (70.6)
No	206 (18.5)	157 (18.0)
N/A (non-solid tumor)	96 (8.6)	96 (11.0)
Unknown	16 (1.4)	12 (1.4)
Chemotherapy, *n* (%)		
Yes	740 (66.3)	562 (64.4)
No	313 (28.1)	252 (28.9)
Unknown	62 (5.6)	58 (6.7)
Radiotherapy, *n* (%)		
Yes	587 (52.6)	487 (55.9)
No	467 (41.9)	337 (38.6)
Unknown	61 (5.5)	48 (5.5)
Hormone therapy for breast cancer (*n*=741), *n* (%)		
Yes	233 (38.9)	128 (27.6)
No	208 (34.7)	172 (37.1)
Unknown	157 (26.2)	164 (35.3)
*Patient-reported outcomes*		
Cognitive functioning, mean (SD)		
EORTC QLQ-C30 (*n*=554)	83.5 (20.7)	80.8 (20.9)
MFI (*n*=1061)	10.4 (4.3)	10.7 (4.4)
CIS (*n*=144)	13.6 (8.4)	12.8 (7.0)
FAQ (*n*=227)	30.9 (26.5)	35.8 (29.1)
Fatigue^a^, mean (SD)	32.1 (22.9)	34.3 (24.4)
Depression, mean (SD)		
HADS (*n*=1218)	4.1 (4.4)	4.7 (4.8)
BSI (*n*=280)	1.6 (2.9)	1.7 (3.0)
SCL-90 (*n*=143)	23.0 (9.1)	21.4 (5.3)
CES-D (*n*=211)	16.4 (5.2)	18.0 (7.2)
Anxiety, mean (SD)		
HADS (*n*=1216)	5.8 (4.7)	6.2 (5.2)
BSI (*n*=280)	1.5 (2.1)	1.7 (3.4)
SCL-90 (*n*=143)	13.8 (5.4)	13.5 (4.2)

^a^Fatigue is assessed by a subscale of European Organisation Research and Treatment of Cancer Quality of Life Questionnaire-Core 30 (EORTC QLQ-C30) (score ranges 0–100: higher score better cognitive functioning)

*MFI* Multidimensional Fatigue Inventory (score ranges 4–20: higher score worse cognitive functioning), *CIS* Checklist Individual Strength (score ranges 8–56: higher score worse cognitive functioning), *FAQ* Fatigue Assessment Questionnaire (score ranges 0–100: higher score worse cognitive functioning), *HADS* Hospital Anxiety and Depression Scale (score ranges 0–21: higher score more anxiety/depressive symptoms), *BSI* Brief Symptom Inventory (score ranges 0–24: higher score more anxiety/depressive symptoms), *SCL-90* Symptom Checklist (score ranges 0–36/48: higher score more anxiety/depressive symptoms), *CES-D* Center for Epidemiologic Studies Depression (score ranges 0–100: higher score more depressive symptoms)

### Included exercise interventions

The included studies were published between 2005 and 2017, and were carried out in the Netherlands [38–41, 44, 46, 51], Australia [30–32, 36], Germany [33, 43, 48, 49], Norway [34], and the USA [35]. Nine of the 16 studies were performed during cancer treatment and the majority of interventions consisted of supervised exercise (81.8%) (Table 3 and Appendix I). The duration of the intervention varied between 10 weeks and 1 year. Most interventions consisted of both resistance and aerobic exercises (66.4%), usually offered twice a week (82.6%) at moderate-vigorous to vigorous intensity (65.7%) for 30–60 min (70.5%). Of the patients allocated to a control group, 55.6% were assigned to a usual care group, 24.5% to a wait-list control, and 19.8% to an attention control group (Table 3).

**Table 3:** Intervention and exercise-related characteristics of individual participants from 16 randomized controlled exercise trials included in the meta-analysis

	All interventions (*n=*1115) *n* (%)^a^	During treatment (*n=*656) *n* (%)^a^	After treatment **(***n=*419) *n* (%)^a^
Intervention characteristics			
Mode of intervention delivery			
Supervised	912 (81.8)	499 (76.1)	373 (89.0)
Unsupervised	203 (18.2)	157 (23.9)	46 (11.0)
Duration of intervention			
≤12 weeks	471 (42.2)	200 (30.5)	271 (64.7)
12–24 weeks	348 (31.2)	248 (37.8)	100 (23.9)
>24 weeks	256 (23.0)	208 (31.7)	48 (11.5)
Unknown	40 (3.6)	-	-
Type of control group^b^			
Usual care	485 (55.6)	329 (65.0)	156 (47.9)
Wait-list control	214 (24.5)	44 (8.7)	170 (52.1)
Attention control	173 (19.8)	133 (26.3)	–
Exercise-related characteristics			
Exercise frequency			
2 times per week	918 (82.3)	499 (76.1)	419 (100.0)
≥5 times per week	197 (17.7)	157 (23.9)	–
Exercise intensity			
Moderate	311 (27.9)	131 (20.0)	180 (43.0)
Moderate-vigorous to vigorous	732 (65.7)	453 (69.1)	239 (57.0)
Unknown	72 (6.5)	72 (11.0)	–
Exercise type			
AE	186 (16.7)	157 (23.9)	29 (6.9)
RE	112 (10.0)	112 (17.1)	-
AE + RE	740 (66.4)	310 (47.3)	390 (93.1)
RE + Impact training	77 (6.9)	77 (11.7)	–
Exercise session duration			
≤30 min	243 (21.8)	157 (23.9)	46 (11.0)
30–60 min	786 (70.5)	499 (76.1)	287 (68.5)
>60 min	86 (7.7)	–	86 (20.5)

^a^Proportion of patients from exercise groups

^b^Proportion of patients from control groups (*n=*872 for all interventions, *n=*506 for interventions during cancer treatment, *n=*326 for interventions after cancer treatment)

*AE* aerobic exercise, *RE* resistance exercise

### Exercise effects on self-reported cognitive functioning and potential moderators

**Fig. 1:**
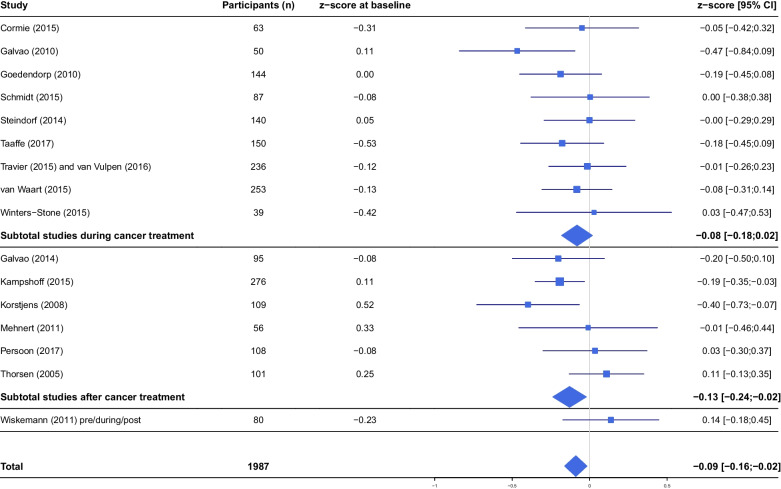
Forest plot of the effect of exercise interventions on self-reported cognitive functioning. Subtotals show the exercise effect in studies that applied the intervention during or after the cancer treatment. *CI*, confidence interval

Overall, exercise interventions had a statistically significant effect of minimal size on self-reported cognitive functioning (β =−0.09, 95% CI −0.16; −0.02) compared to controls (Table 4, Fig. 1). Larger, although still minimal, effects were observed in exercise interventions that were delivered after cancer treatment (β = −0.13, 95% CI −0.24; −0.02) or had an intervention duration of 12 weeks or shorter (β = −0.14, 95% CI −0.25; −0.04) or longer than 24 weeks (β = −0.18, 95% CI −0.32; −0.02). None of the sociodemographic, clinical, or other intervention characteristics significantly moderated exercise effects on self-reported cognitive functioning (Table 4). Baseline anxiety moderated the exercise intervention effect on self-reported cognitive functioning (*p*=0.08). Participants with low baseline anxiety experienced larger effects (β = −0.10, 95% CI −0.19; −0.02) on cognitive functioning compared to participants with higher baseline anxiety (β = 0.07, 95% CI −0.12; 0.26) (Table 4).

**Table 4:** Effects and moderators of the effects of exercise on self-reported cognitive functioning

Self-reported cognitive functioning	*z*-score	
	β (95% confidence interval)	*p***-**value LRT
Overall exercise effect (*n*=1987)	**−0.09 (−0.16; −0.02)**	
Exercise effect after treatment (*n*=745)	**−0.13 (−0.24; −0.02)**	
Exercise effect during treatment (*n*=1162)	**−**0.08 (**−**0.18; 0.02)	
*Sociodemographic moderators*		
Age	No significant interaction	0.17
Education level	No significant interaction	0.73
Gender	No significant interaction	0.32
*Clinical moderators*		
Chemotherapy	No significant interaction	0.43
Hormone therapy (breast cancer)	No significant interaction	0.12
Cancer type	No significant interaction	0.32
*Baseline levels of patient-reported outcomes*		
Baseline fatigue	No significant interaction	0.38
Baseline cognitive functioning	No significant interaction	0.60
Depression*	No significant interaction	1.00
Anxiety^a,^*		0.08
Yes (*n*=325)	0.07 (**−**0.12; 0.26)	
No (*n*=1314)	**−0.10 (−0.19; −0.02)**	
*Intervention moderators*		
Delivery mode^b^		0.97
Unsupervised	Reference	
Supervised	**−**0.004 (**−**0.15; 0.14)	
Intervention length		0.07
=<12 weeks	**−0.14 (−0.25; −0.04)**	
12–24 weeks	0.01 (**−**0.13; 0.15)	
>24 weeks	**−0.18 (−0.32; −0.02)**	

^a^Originally, anxiety was measured on a continuous scale. The score was dichotomized using cutoff scores from literature: HADS-A >9 [52], or using the mean: BSI and SCL-90

^b^Interaction testing is not applicable; therefore, differences between subgroups are reported. LRT of the model including the intervention characteristic vs the main model is presented

*LRT* likelihood ratio test

The respective *z*-scores are calculated using the MFI, CIS, FAQ and EORTC QLQ C30 questionnaire

A negative *z*-score indicates an improvement in cognitive functioning

*Excluding two studies [31, 35] which did not measure depression or anxiety

### Sensitivity analysis

In post-treatment exercise intervention studies (*n*=745), we found a minimal, significant exercise effect on self-reported cognitive functioning in patients with cognitive problems at baseline (*n*=611, 82%) (β=−0.19, 95% CI −0.31; −0.06).

## Discussion

Based on this IPD meta-analysis of 16 RCTs, including 1987 patients with multiple types of cancer, we found that exercise has a minimal, beneficial effect on self-reported cognitive functioning, with slightly larger effects being observed when the exercise intervention is delivered post-treatment or had an intervention length of 12 weeks or shorter or 24 weeks and longer. These benefits were consistent across subgroups based on clinical or sociodemographic characteristics. Larger, but still minimal, effects on cognitive functioning were found in participants with low levels of anxiety and in patients with more cognitive problems at baseline who exercised after treatment.

The minimal, beneficial effect of exercise on self-reported cognitive functioning that we found in this study is smaller compared to the effect size reported in the Physical Activity and Memory (PAM) study, which is, to date, the only sufficiently powered study to assess exercise effects on cognitive functioning [22]. The PAM study focused on patients with breast cancer who still reported cognitive problems 2–4 years after completion of chemotherapy that were confirmed by objective neuropsychological testing [53]. In the majority of studies included in this IPD meta-analysis, cognitive functioning was not the primary outcome and no screening for cognitive problems was performed. Hence, the present study sample differs from the PAM sample and includes patients with relatively low levels of cognitive problems and little room for improvement. Consequently, the true potential of exercise to remediate self-reported cognitive functioning might be underestimated in the current study (ES=0.43 in the PAM study vs. ES=0.13 in our study). Our sensitivity analysis, which only included patients with room for improvement (i.e., patients with cognitive problems), showed larger, but still minimal, exercise effects (ES=0.19). Furthermore, our study population received an exercise program during cancer treatment or shortly after finishing cancer treatment, whereas exercise perhaps is more likely to be effective in patients who report persistent cognitive problems.

The use of IPD for our analysis offered the unique opportunity to investigate whether exercise effects differ across subgroups of patients. In previous studies in breast cancer survivors, endocrine therapy and time since surgery (≤2 years vs. ˃2 years) were found to be statistically significant moderators of exercise effects on cognitive functioning [22, 54]. In line with our results, no other significant sociodemographic or clinical moderators were identified, suggesting that exercise might be helpful for improving cognitive functioning regardless of such characteristics. Importantly, these stand-alone RCTs were not powered to detect moderators of exercise effects. It is for this reason that IPD meta-analysis has been recommended as the preferred method to identify moderators of treatment effects [25, 26].

Some impact of exercise interventions on self-reported cognitive functioning was observed in patients without anxiety symptoms, whereas non-significant negative effects of exercise were found in patients with anxiety symptoms. We hypothesize that the cause of subjective cognitive problems might differ between patients with and without anxiety symptoms. A previous study in breast cancer survivors after chemotherapy showed that heightened baseline levels of anxiety symptoms are associated with self-reported cognitive problems [55]. Given the complex nature of anxiety, exercise alone might be less effective for improving cognitive functioning in patients experiencing anxiety symptoms. In addition to exercise, other treatment approaches targeting anxiety (e.g., cognitive-behavioral therapy) might be necessary to improve cognitive functioning in patients with anxiety symptoms. It is also possible that patients who experience anxiety symptoms might be less compliant with an exercise program [56].

Since cognitive functioning was often reported as a secondary or exploratory outcome in the studies included in this IPD meta-analysis, brief questionnaires, measuring only one or two domains of self-reported cognitive functioning, were used in all included studies. A tool, specifically designed to measure self-reported cognitive problems, such as the FACT-Cog questionnaire, might help to better capture cognitive problems in future studies, ideally complemented by objective measures of cognitive functioning which are commonly described as golden standard [57]. Of note, objective and subjective measures of cognitive functioning are generally weakly correlated since many self-reported cognitive functioning measures are multidimensional and capture psychosocial and emotional symptoms as well [57]. Therefore, in future exercise oncology studies, we would recommend the use of a full neuropsychological test battery in addition to a self-report questionnaire in order to investigate cognitive problems in patients with cancer in a comprehensive manner. Two of the RCTs in this IPD meta-analysis included objectively assessed cognitive functioning as a secondary outcome [48, 49]. Both studies, which were conducted in patients during breast cancer treatment, found that objective cognitive performance improved slightly more in the exercise group than in the control group. However, between-group differences were not significant post-intervention. In addition, they did not find a significant effect on self-reported cognitive functioning post-intervention when comparing the exercise group to the control group. Three small pilot studies in patients after completion of cancer treatment, which were not included in the current IPD meta-analysis, applied neuropsychological testing and found mixed effects of physical exercise on tested cognitive functioning [54, 58, 59]. In the aforementioned PAM study, tested cognitive functioning was not affected by the exercise intervention, except in highly fatigued patients [22].

The current study is the first to summarize, pool, and analyze IPD of 16 RCTs, including almost 2000 patients during and after cancer treatment, to investigate exercise effects on self-reported cognitive functioning. A major strength of this review is the availability of a large amount of IPD, enabling us to investigate a range of potential sociodemographic and clinical patient-level moderators. Another strength is that we carefully standardized outcome data and used uniform statistical techniques across all studies. However, several limitations of our study should also be mentioned. First, as stated above, the use of brief questionnaires (measuring one or two domains of cognitive functioning) to measure self-reported cognitive functioning might have resulted in less measurement responsiveness. This stresses the importance of including more comprehensive instruments to assess self-reported cognitive functioning. Second, in the current IPD meta-analysis, we included all studies available in the POLARIS database. However, since this database originally comprises RCTs, which primarily investigate exercise effects on HRQoL, not all available RCTs investigating exercise effects on cognitive functioning were included in this analysis. As already mentioned, cognitive functioning was not the primary outcome in the included studies and no screening for cognitive problems was performed, resulting in low levels of cognitive problems at baseline. Therefore, the results of this study should be considered exploratory. Nevertheless, this meta-analysis provided sufficient statistical power to gain more insight into exercise effects on self-reported cognitive functioning and to determine whether these effects differ between subgroups. The results can inform the design of future studies in this field. Third, this study included heterogeneous patient populations (e.g., cancer type, stage, and timing of treatment), intervention types, intensity and duration, and outcome measures, which impedes the generalizability of our findings. Although the number of participants included in this analysis was considerable, the statistical power was insufficient to detect intervention- and exercise-related moderators, since these variables are defined at the study level, resulting in little variation across studies. Furthermore, patients who are willing to participate in exercise intervention trials are often highly motivated to be physically active. As a result, this selection of patients could have impacted baseline levels of cognitive functioning, leading to ceiling effects and hampering the generalizability of the results. Finally, adherence to the exercise program and the extent of contamination in the control group was unknown in the majority of the included studies, both of which could have an impact on the observed effects.

## Conclusion

In conclusion, we found a statistically significant, but minimally beneficial effect of exercise on self-reported cognitive functioning in patients with cancer who were not specifically selected for experiencing cognitive problems at baseline. Consistent minimal, beneficial effects were observed across subgroups of patients with different sociodemographic and clinical characteristics. Slightly larger exercise effects on cognitive functioning were observed in post-cancer treatment studies, studies with an intervention length of 12 weeks or shorter or 24 weeks and longer and in patients without anxiety. While the study sample was large and based on individual-level data, it was also highly heterogeneous with respect to diagnoses, disease stages, and exercise characteristics. To date, due to insufficient evidence, the current exercise guidelines for cancer survivors do not include any exercise prescriptions to improve self-reported cognitive functioning. Our study provides some evidence in support of including cognitive symptoms as a target for exercise interventions. However, sufficiently powered and properly designed trials in more homogenous populations of cancer patients are warranted in order to make more definitive recommendations and include these recommendations in the exercise guidelines for cancer survivors.

## Supplementary information

ESM 1 (DOCX 16 kb)

## Data Availability

A request can be sent to the corresponding author to collaborate with the POLARIS Consortium.
