# Supplementary material for: Moderators of exercise effects on self-reported cognitive functioning in cancer survivors: an individual participant data meta-analysis
Source: J Cancer Surviv. 2023 May 9;18(5):1492–503. doi: 10.1007/s11764-023-01392-3 (PMC11424665; doi:10.1007/s11764-023-01392-3)
Supplement: Supplementary file 1 — (DOCX 16 kb) [file 11764_2023_1392_MOESM1_ESM.docx]

| **Appendix I**. Descriptive characteristics of studies evaluating the effects of exercise interventions on self-perceived cognitive functioning (*n*=16) | | | | | | | | | | | | |
| --- | --- | --- | --- | --- | --- | --- | --- | --- | --- | --- | --- | --- |
|  | | | | | **Intervention** | | | **Exercise** | **Control** | **Questionnaires** | | |
| **Author (year)**  **Acronym** | **Country** | **N** | **Age, mean (SD)** | **Diagnosis** | **Timing** | **Delivery mode** | **Duration (weeks)** | **FITT** |  | **Cognitive functioning** | **Fatigue** | **Anxiety/**  **depression** |
| Cormie (2015) | AUS | 64 | 68 (7.2) | Prostate | During ADT | Supervised | 12 | F: 2/week  I: moderate-vigorous  T: RE+AE  T: 60 min | Usual care | EORTC QLQ-C30 | FACT-fatigue | BSI-18 |
| Galvão (2010) | AUS | 57 | 70 (7.2) | Prostate | During ADT | Supervised | 12 | F: 2/week  I: moderate T: RE+AE  T: 60 min | Usual care | EORTC QLQ-C30 | EORTC QLQ-C30 fatigue | N/A |
| Galvão (2014)  *RADAR* | AUS | 100 | 72 (6.5) | Prostate | Post | Supervised | 26 | F: 2/week I: moderate-vigorous  T: RE+AE  T: 60 min | Usual care with PA brochure | EORTC QLQ-C30 | EORTC QLQ-C30 fatigue | BSI |
| Goedendorp (2010) | NL | 144 | 57 (10.5) | Mixed | During | Home-based | Mean: 31.7 | F: towards 5/week  I: ?  T: AE  T: towards 60 min | Usual care | CIS | CIS | SCL-90 |
| Kampshoff (2015) *REACT* | NL | 277 | 54 (11.1) | Mixed | Post | Supervised | 12 | F: 2/week  I: moderate vs.   vigorous  T: RE+AE  T: 60 min | Wait-list | MFI | MFI | HADS |
| Korstjens (2008) *Oncorev* | NL | 133 | 51 (10.2) | Mixed | Post | Supervised | 12 | F: 2/week  I: AE: moderate-  vigorous  RE: low-moderate  T: RE+AE  T: 120 min | Wait-list | MFI | MFI | HADS |
| Mehnert (2011) | GER | 58 | 52 (8.4) | Breast | Post | Supervised | 10 | F: 2/week  I: moderate  T: AE + gymnastics +   movement games +   relaxation  T: 90 min | Wait-list | EORTC QLQ-C30 | EORTC QLQ-C30 fatigue | HADS |
| Persoon (2017)  *EXIST* | NL | 109 | 53 (10.8) | Haematological | Post SCT | Supervised | 18 | F: 2/week  I: moderate-vigorous  T: RE+AE  T: 60 min | Usual care | MFI | MFI | HADS |
| Schmidt (2015)  *BEATE* | GER | 87 | 53 (10.0) | Breast | During CT | Supervised | 12 | F: 2/week  I: moderate-vigorous  T: RE  T: 60 min | Attention control | FAQ | FAQ | CES-D |
| Steindorf (2014)  *BEST* | GER | 140 | 56 (8.9) | Breast | During RT | Supervised | 12 | F: 2/week  I: moderate-vigorous  T: RE  T: 60 min | Attention control | FAQ | FAQ | CES-D |
| Taaffe (2017) | AUS | 150 | 69 (9.0) | Prostate | During ADT | Supervised | 26 | F: 2/week  I: moderate-vigorous  T: RE T: 60 min | Wait-list | EORTC QLQ-C30 | EORTC QLQ-C30 fatigue | BSI-18 |
| Thorsen (2005) | NOR | 139 | 40 (8.1) | Mixed | Post | Unsupervised | 14 | F: 2/week or more  I: moderate-vigorous  T: RE+AE  T: aim 30 min | Usual care | EORTC QLQ-C30 | EORTC QLQ-C30 fatigue | HADS |
| Travier (2015), van Vulpen (2016) *PACT* | NL | 237 | 51 (8.8) | Breast and colorectal | During CT | Supervised | 18 | F: 2/week  I: moderate-vigorous  T: RE+AE T: 60 min | Usual care | MFI | MFI | HADS |
| Van Waart (2015)  *PACES* | NL | 253 | 51 (9.5) | Breast and colorectal | During CT | Supervised vs. unsupervised | Mean: 15.9 | F: supervised 2/week  Unsupervised   towards 5/week  I: supervised:   moderate- vigorous  unsupervised:   moderate  T: supervised: RE+AE  unsupervised: AE T: supervised: 60 min  unsupervised: aim   30 min | Usual care | MFI | MFI | HADS |
| Winters-Stone (2015) | USA | 51 | 70 (8.5) | Prostate | During ADT | Supervised | 52 | F: 2/week supervised   (+1/week   unsupervised)  I: moderate T: RE+impact T: 60 min | Attention control | EORTC QLQ-C30 | SCFS-6 | N/A |
| Wiskemann (2011) | GER | 80 | 48 (14.4) | Haematological | Pre/during/post | Supervised | Median exercise: 16.4  Control: 15.7 | F: 5/week  I: moderate-vigorous  T: RE+AE  T: AE: 20-40 min | Attention control | MFI | MFI | HADS |
| Descriptive characteristics are based on the data in the POLARIS database. Abbreviations: SD=standard deviation; ADT= androgen deprivation therapy; CT=chemotherapy; RT=radiotherapy; SCT=stem cell transplantation; AE=aerobic exercise; RE=resistance exercise; FACT‐fatigue=Functional assessment of cancer treatment fatigue scale; EORTC QLQ-C30 (fatigue)= European Organization for Research and Treatment of Cancer Quality of Life Questionnaire – Core 30 (fatigue subscale); CIS=Checklist individual strength; MFI=Multidimensional fatigue inventory; FAQ=Fatigue Assessment Questionnaire; SCFS‐6=Schwartz Cancer Fatigue Scale; HADS=Hospital Anxiety and Depression Scale; BSI=Brief Symptom Inventory; CES-D=Center for Epidemiological Studies Depression Scale. | | | | | | | | | | | | |
